# Supplementary material for: Retinal microvasculature and cerebral small vessel disease in the Lothian Birth Cohort 1936 and Mild Stroke Study
Source: Sci Rep. 2019 Apr 19;9:6320. doi: 10.1038/s41598-019-42534-x (PMC6474900; doi:10.1038/s41598-019-42534-x)
Supplement: Supplementary file 1 — Supplementary Material [file 41598_2019_42534_MOESM1_ESM.docx]

**Supplementary material**

Retinal microvasculature and cerebral small vessel disease in the Lothian Birth Cohort 1936 and Mild Stroke Study

Sarah McGrory^a,b*^, Lucia Ballerini^a^, Fergus N. Doubal^a^, Julie Staals^c,d^, Mike Allerhand^b,e^, Maria del C. Valdes-Hernandez^a^, Xin Wang^a^, Tom MacGillivray^a^, Alex S.F. Doney^f^, Baljean Dhillon^a^, John M. Starr^e,g^, Mark E. Bastin^e,h^, Emanuele Trucco^i^, Ian J. Deary^b,e**,^ Joanna M. Wardlaw^,e,h,j**^

^a^ VAMPIRE project, Centre for Clinical Brain Sciences, University of Edinburgh, Edinburgh, UK

^b^ Department of Psychology, University of Edinburgh, Edinburgh, UK

^c^ Department of Neurology, Maastricht University Medical Center, Maastricht, The Netherlands

^d^ Cardiovascular Research Institute Maastricht (CARIM), Maastricht University, Maastricht, The Netherlands

^e^ Centre for Cognitive Ageing and Cognitive Epidemiology, University of Edinburgh, Edinburgh, UK

^f^ Division of Cardiovascular and Diabetes Medicine, Medical Research Institute, Ninewells Hospital and Medical School, Dundee, UK

^g^ Alzheimer Scotland Dementia Research Centre, University of Edinburgh, Edinburgh, UK

^h^ Scottish Imaging Network, a Platform for Scientific Excellence (SINAPSE) Collaboration, Edinburgh, UK

^i^ VAMPIRE project, Computing, School of Science and Engineering, University of Dundee, Dundee, UK

^j^ UK Dementia Research Institute at the University of Edinburgh, Chancellor’s Building, Edinburgh, UK

* Corresponding author

** These authors contributed equally.

**Retinal measurement and data reduction**

**Retinal measurements**

VAMPIRE (version 3.1) analysis yielded 143 retinal vasculature measurements from the width, tortuosity and branching geometry of the retinal vessels of each eye (286 for both right and left eyes) (see Supplementary Table S2 for description of retinal measurements). Separate arteriolar and venular measures were calculated and are indicated by lowercase ‘a’ or ‘v’. Measurements zones within which to measure retinal parameters are set in relation to the centre of the optic disc (OD) and its size (see Supplementary Fig. S1 for details of measurement zones). Briefly, vessel width measurements are derived from within Zone B, branching geometry parameters and fractal dimension are measured from vessels within Zone C and various tortuosity measurements are calculated within Zone C and spanning the retinal image.

**Retinal data reduction**

The data reduction described here relates to LBC1936. In order to reduce the numerous variables to a manageable number, where possible we attempted to extract latent variables reflecting shared variance between variables. This process of data reduction is illustrated in Supplementary Fig. S2.

*Measurements from left and right eyes*

All left and right eye measurements were averaged to provide 143 mean retinal measurements. While averaging left and right measurements may attenuate effects which might be detected when using one eye only, we chose to do so in order to reduce the number of variables, reduce multicollinearity and increase reliability.

A thorough analysis of agreement between measurements from right and left eyes is lacking. Most studies have focused on analysis of vessel diameters, with good correlation reported between right and left vessel diameter measurements (*r* > 0.70)^1-4^. Moderate correlations between fractal dimension of right and left eyes have been found (*r* = 0.403-0.582)^5,6^. While analysis of the other retinal variables included in the current study are limited, MacGillivray et al. (2015) found significant positive but low (< 0.25) correlations for tortuosity, branching coefficient and branching angle^7^.

While interocular symmetry remains largely unresolved, commonly, a prespecified eye (usually the right eye) is used for the measurement and analysis, which is replaced with the measurement from the other eye (usually the left eye) only if the image of the first eye was not usable or is of inferior quality. Some studies including the Atherosclerosis Risk in Communities Study (ARIC) chose to image half of the participants’ right eyes and half of the participants’ left eyes^8-13^. These practices of combined analysis of either eye could also result in attenuation of potential effects found when using one eye only. Furthermore, several studies have also taken the mean of both eyes for analysis^14-16^.

We do note that further work on interocular symmetry and the impact of asymmetry on determining associations with brain imaging features is necessary. Examining associations between retinal measurements from each eye and SVD should be investigated in future research.

*Tortuosity*

100 tortuosity measurements (50 arteriolar, 50 venular) were estimated from each image. See Supplementary Table S2 and Supplementary Fig. S3-S5 for details of measurements. Correlations between tortuosity variables (Supplementary Tables S3a & b) demonstrate, predictably, strong associations between the various measurements of tortuosity. The tortuosity of vessels is calculated using the same algorithm for all variables^17^. The variables differ only by the vessels identified for measurement and the zone and quadrant of the image where they are measured.

Four parameters (torta, tortv, tortImageG1a and tortImageG1v) estimate the tortuosity of arteriolar and venular paths across the retinal image. Torta and tortv estimations are based on the tortuosity of the six largest arterioles and venules within Zone C, respectively. TortImageG1a and tortImageG1v provide a more global estimation of tortuosity calculated for entire vessel paths that span from Zone B to Zone C.

The remaining variables measure tortuosity within quadrants of the retinal image (Q1-Q4, see Supplementary Fig. S3). Descriptions are provided in Supplementary Table S2. Briefly, tortuosity is estimated from the weighted average of the tortuosity of (a) all arterioles/venules in quadrants 1-4 (tortQ1-Q4a/v); (b) all generation 1 arterioles/venules in quadrants 1-4 (tortQ1-Q4G1a/v; Supplementary Fig. S4); (c) the main arteriolar/venular path in quadrants 1-4 (tortMainPathQ1-Q4a/v; Supplementary Fig. S5).

In each case, principal components analysis (PCA) was used to examine the variance shared by the four quadrants and to provide scores on a latent tortuosity trait. Measurements based on the different groups of quadrant were entered into PCA separately. The scree slope and eigenvalues >1 criteria were used to assess whether a single latent trait of tortuosity might be extracted from the measures. In each case, the results indicated a clear one-factor solution (see Supplementary Fig. S6), with the first unrotated factor explaining between 39% and 58% of the variance. Supplementary Table S4 shows the loadings of each measurement on the first unrotated principal component. All measures had high loadings (ranging between .827 and .544). Scores on these first unrotated principal components were saved as standardised scores.

The correlations between these scores, Zone C tortuosity measurements (torta and tortv), and whole-image tortuosity (tortImageG1a and tortImageG1v) were assessed (Supplementary Tables 4a & b). PC scores correlated strongly with each other and with the measured tortuosity variables. The correlations indicate a considerable degree of shared variance between the components and measured variables. The quadrant-based components did not appear to contribute additional information to the measured tortuosity variables. tortImageG1a and tortImageG1v, as indicators of tortuosity across a larger area than measurements in Zone C (torta and tortv), were selected as appropriate measures for analysis. These are described as TORTa and TORTv hereafter, and in the manuscript.

*Fractal dimension*

Three different multifractal dimensions of both arteriolar and venular networks; D0, D1 and D2, were calculated^18^. These measurements are referred to as the capacity dimension, the entropy dimension and the correlation dimension, respectively. As correlations between measurements were very strong (*r* > .98), any one of these measures could be chosen to represent the complexity of the vascular tree. D0 was selected for measurement of both the arteriolar and venular fractal dimension (D0a and D0v, denoted hereafter, and in the manuscript as arteriolar [FDa] and venular [FDv] fractal dimension).

*Vessel width*

Five measurements relate to vessel calibre: central retinal arterial (CRAE) and venular (CRVE) equivalent calibre, arteriole-venular ratio (AVR), standard deviation of the arteriolar (BSTDa) and venular (BSTDv) width in Zone B.

AVR was originally conceived as a measure of arteriolar narrowing^19^, with smaller AVR reflecting narrower arterioles relative to presumed stable venular widths. However, AVR fails to take into account independent changes in arteriolar and venular calibre and may be influenced by concurrent arteriolar and venular changes^20-21^. Furthermore, as a ratio of the calibre of arterioles to venules, the information will be provided by its components, CRAE and CRVE. Therefore, AVR was excluded leaving CRAE and CRVE, along the variation in vessel calibre measured by BSTDa and BSTDv, for measurement of the arteriolar and venular calibres.

*Vessel width estimation methods*

16 measurements were calculated using two different methods of width estimation: “Hermite”, using a general model of the intensity profile across vessels based on a Hermite polynomial^22^, and “Spline”, based on an approximation of vessel boundaries with a pair of splines constrained to be parallel^23^. These algorithms enable the calculation of 32 variables quantifying the branching geometry and vessel width gradients.

Correlations between measurements calculated using the two different methods were poor (Supplementary Tables S6a & b). The correlations of variables based on vessel boundaries identified with the Hermite/Spline methods with other retinal variables were examined to determine which method of width estimation produced variables leading to the most effective data reduction when considered with other retinal measurements. (Supplementary Tables S7a & b).

The Spline method was chosen for the width gradient measurements (arteriolar: GradQ1a, GradQ2a, GradQ3a, GradQ4a; and venular: GradQ1v, GradQ2v, GradQ3v and GradQ4v). The Hermite method was chosen for arteriolar and venular branching measurements (branching coefficient [BC], junctional exponent [JE], asymmetry factor [AF], length diameter ratio [LDR]).

*Branching geometry measurements*

Two of the measurements of branching geometry, namely branching coefficient and junctional exponent, were strongly correlated (*r* > .9), indicating that only one of these measurements would be required. Junctional exponent was excluded from further analysis.

Arteriolar and venular branching coefficient (BCa, BCv), asymmetry factor (AFa, AFv) and length-diameter ratio (LDRa, LDRv) were retained for analysis.

*Gradient measurements*

Principal components analysis was carried out on the measurements for arteriolar (Gradient Q1a, Gradient Q2a, Gradient Q3a, GradientQ4a) and venular (Gradient Q1v, Gradient Q2v, Gradient Q3v, GradientQ4v) width gradient measurements. The number of components extracted was determined by examination of eigenvalues and scree plots (Supplementary Fig. S7). Eigenvalues and examination of the scree plot for the arteriolar gradient measurements suggested the extraction of one component. This first unrotated component accounted for 33% of the total variance.

Eigenvalues and the scree plot for venular gradient measurements suggested the extraction of two components. The first accounted for 31.9% of the variance, and the second accounted for 25.1%. These components were rotated using an oblique rotation (direct oblimin) to obtain a more interpretable factor structure. Supplementary Table S8 show the loadings of each arteriolar and venular measurement on the first unrotated principal component and the loadings on the two venular factors after rotation. For comprehensibility, these components are referred to as ‘factors’.

This process of data reduction left 14 measured variables (TORTa, TORTv, FDa, FDv, CRAE, CRVE, BSTDa, BSTDv, BCa, BCv, AFa, AFv, LDRa and LDRv) and two factors (arteriolar width gradient and venular width gradient) for further analysis. Correlations between variables are presented in Supplementary Table S9. A visual representation of these measurements is presented in Supplementary Fig. S8.

Correlations between CRAE, CRVE and arteriolar and venular fractal dimension suggest shared variance among the measurements. This was confirmed by principal components analysis, in which the first unrotated component accounted for 54% of the total variance

Supplementary Table S10 shows the loadings of each measurement on the first unrotated principal component.

Supplementary Table S1. Sensitivity analysis excluding those with history of stroke or imaging evidence of stroke (*n* =84) in the Lothian Birth Cohort 1936

|  |  |  |  |  |  |  |  |  |  |  |  |  |  |  | Gradient a | Gradient v |
| --- | --- | --- | --- | --- | --- | --- | --- | --- | --- | --- | --- | --- | --- | --- | --- | --- |
|  | CRAE | CRVE | BSTDa | BSTDv | FDa | FDv | TORTa | TORTv | BCa | BCv | AFa | AFv | LDRa | LDRv | 1^st^  Unrotated PC | 1^st^  Unrotated PC |
|  | β | β | β | β | β | β | β | β | β | β | β | β | β | β | β | β |
| WMH % in BTV* | -.091 | **-.125^a^** | .038 | .022 | **-.205***** | **-.175*** | -.011 | .071 | .016 | -.057 | .035 | .081 | .095 | .069 | -.006 | .043 |
| BTV mm^3^ | -.087 | .049 | -.095 | -.074 | .000 | -.013 | .006 | -.081 | .023 | -.042 | -.065 | .003 | .003 | -.001 | -.025 | .060 |
| WMH % in ICV* | -.092 | **-.125^a^** | .038 | .023 | **-.206***** | **-.178*** | -.010 | .070 | .017 | -.056 | .037 | .079 | .100 | .074 | -.006 | .042 |
| ICV mm^3^ | -.092 | .082 | -.108 | -.105 | .017 | .051 | -.004 | -.044 | -.005 | -.114 | -.125 | .039 | -.111 | -.116 | -.038 | .099 |
| Fazekas Periventricular | **-.099^a^** | -.047 | .056 | .037 | **-.176***** | **-.155^a^** | .061 | .049 | -.071 | -.044 | .029 | .074 | .023 | .040 | -.049 | -.032 |
| Fazekas Deep | -.001 | -.061 | .059 | .064 | **-.104^a^** | **-.121^a^** | .023 | **.116^a^** | -.024 | -.009 | .034 | .010 | .089 | .072 | -.043 | .093 |
| Atrophy Deep | -.046 | -.001 | .014 | -.003 | -.051 | .069 | .012 | **.111^a^** | .092 | .067 | -.102 | .022 | -.055 | -.047 | -.009 | .008 |
| Atrophy Superficial | -.027 | .005 | .018 | -.038 | -.046 | .060 | .017 | .078 | .087 | .072 | -.093 | .030 | -.090 | -.081 | -.070 | .020 |
| PVS | -.017 | -.003 | .005 | -.027 | -.002 | .023 | .015 | **.135^a^** | -.011 | .000 | .034 | .020 | -.003 | .053 | -.042 | -.026 |
| Microbleeds | -.037 | -.026 | -.045 | .066 | .012 | -.075 | .035 | -.043 | -.007 | -.024 | -.029 | .081 | .022 | .010 | -.029 | -.036 |
| SVD score | -.010 | -.018 | .010 | -.009 | -.025 | -.007 | .029 | **.143^a^** | -.039 | -.024 | .036 | .016 | .049 | .088 | -.052 | -.006 |

*Note.* Model adjusted for age and sex. WMH=white matter hyperintensities; BTV=brain tissue volume; ICV=intracranial volume; PVS= basal ganglia perivascular spaces; SVD=small vessel disease; CRAE=central retinal artery equivalent; CRVE=central vein retinal equivalent; BSTDa=standard deviation of arteriolar widths in Zone B; BSTDv=standard deviation of venular widths in Zone B; FDa= fractal dimension arteriolar; FDv=fractal dimension venular; TORTa= arteriolar tortuosity; TORTv=venular tortuosity; BCa=arteriolar branching coefficient; BCv=venular branching coefficient; AFa=arteriolar asymmetry factor; AFv=venular asymmetry factor; LDRa=length-diameter ratio arteriolar; LDRv=length-diameter ratio venular; PC=principal component. Bolded values are statistically significant. WML % volumes and tortuosity log transformed. β, Standardised regression coefficients

* = *p* < .05; ** = *p* < .01; *** = *p* < .001; all p-values corrected for False Discovery Rate. ^a^ = value was statistically significant at *p* < .05 before FDR correction

Supplementary Table S2. Description of retinal parameters with relevant retinal zone

| Parameter | Description | Retinal Zone | Summary |
| --- | --- | --- | --- |
| **CRAE** | Central Retinal Artery Equivalent | B | Summary measure of vessel width-the 6 largest vessels within Zone B are measured  AVR is the ratio of CRAE and CRVE originally considered a measure of arteriolar narrowing^20^ |
| **CRVE** | Central Retinal Vein Equivalent | B |  |
| AVR | Arteriole-venular ratio  CRAE/CRVE=AVR | B |  |
| **D0a** | Fractal (capacity) dimension of arteriolar network | C | A measure of the degree of branching complexity of the retinal vasculature.  Generalised sandbox method was used to calculate multifractal dimensions D_0_, D_1_ and D_2_^18^ |
| D1a | Fractal (information) dimension of arteriolar network | C |  |
| D2a | Fractal (correlation) dimension of arteriolar network | C |  |
| **D0v** | Fractal (capacity) dimension of venular network | C |  |
| D1v | Fractal (information) dimension of venular network | C |  |
| D2v | Fractal (correlation) dimension of venular network | C |  |
| **BCa** | Branching coefficient arteriole | C | The ratio of the sum of the cross-sectional areas of the two daughter vessels to the cross-sectional area of the parent vessel at an arteriolar bifurcation.  Calculated twice, with different techniques to estimate vessel widths: “Hermite”^22^ and “Spline”^23^ |
| **BCv** | Branching coefficient venule | C |  |
| **AFa** | Asymmetry factor arteriole | C | The cross sectional area of the minor daughter divided by that of the major.  Calculated twice using Spline and Hermite techniques. |
| **AFv** | Asymmetry factor venule | C |  |
| **LDRa** | Length diameter ratio arteriole | C | Vessel length from the midpoint of one vascular bifurcation to the midpoint of the next bifurcation, expressed as a ratio to the diameter of the parent vessel at the first bifurcation.  Calculated twice using Spline and Hermite techniques. |
| **LDRv** | Length diameter ratio venule | C |  |
| JEa | Junctional exponent arteriole | C | Expresses the deviation from optimality of the ratio of vessel widths at a bifurcation.  Calculated twice using Spline and Hermite techniques. |
| JEv | Junctional exponent venule | C |  |
| **BSTDa** | Standard deviation of arteriolar width in Zone B | B | Standard deviation of the widths of the arterioles used in the CRAE calculation |
| **BSTDv** | Standard deviation of venular width in Zone B | B | Standard deviation of the widths of the venules used in the CRVE calculation |
| **GradQ1-Q4a** | Width gradient arteriole | C | Gradient of the width of the main arteriolar path in each quadrant 1 to 4.  Calculated twice using Spline and Hermite techniques |
| **GradQ1-Q4v** | Width gradient venule | C | Gradient of the width of the main venular path in each quadrant 1 to 4.  Calculated twice using Spline and Hermite techniques |
| torta | Tortuosity | C | Calculated from the 6 largest arterioles and venules in Zone C^17^  (Average, minimum and maximum values also measured) |
| tortv |  | C |  |
| **tortImageG1a** |  | Calculated for entire vessel paths that span from Zone B to Zone C | Weighted average* of the tortuosity of all arterioles in the entire image (Median, standard deviation, minimum and maximum values also measured) |
| **tortImageG1v** |  | Entire vessel path | Weighted average of the tortuosity of all venules in the entire image (Median, standard deviation, minimum and maximum values also measured) |
| tortQ1-Q4a |  | Entire vessel path | Weighted average of all arteriolar paths in quadrants 1 to 4 (Median, standard deviation, minimum and maximum values also measured) |
| tortQ1-Q4v |  | Entire vessel path | Weighted average of all venular paths in quadrants 1 to 4 (Median, standard deviation, minimum and maximum values also measured) |
| tortQ1-Q4G1a |  | Entire vessel path | Weighted average of all generation 1 ‡ (G1) arterioles in quadrants 1 to 4 (Median, standard deviation, minimum and maximum values also measured) |
| tortQ1-Q4G1v |  | Entire vessel path | Weighted average of all generation 1 (G1) venules in quadrants 1 to 4 (Median, standard deviation, minimum and maximum values also measured) |
| tortMainPathQ1-Q4a |  | Entire vessel path | Weighted average of tortuosity of the main arteriolar path in quadrants 1 to 4 |
| tortMainPathQ1-Q4v |  | Entire vessel path | Weighted average of tortuosity of the main venular path in quadrants 1 to 4 |

*Note.* Bolded variables were selected for analysis. * Weighted average estimates combined average tortuosity from different paths by calculating an average weighted according to the length of the different paths. ‡ G1 vessels are determined automatically by compromising length and curvature of all possible paths connecting the root point and all end points of a vessel tree.

Supplementary Table S3a. Mean arteriolar tortuosity correlations in the Lothian Birth Cohort 1936

|  | torta | Tort  ImageG1a | Tort  MainPath  Q1a | Tort  MainPath  Q2a | Tort  MainPath  Q3a | Tort  MainPath  Q4a | Tort  Q1a | Tort  Q2a | Tort  Q3a | Tort  Q4a | Tort  Q1G1a | Tort  Q2G1a | Tort  Q3G1a | Tort  Q4G1a |
| --- | --- | --- | --- | --- | --- | --- | --- | --- | --- | --- | --- | --- | --- | --- |
| torta | 1 |  |  |  |  |  |  |  |  |  |  |  |  |  |
| tortImageG1a | .716^**^ | 1 |  |  |  |  |  |  |  |  |  |  |  |  |
| tortMainPathQ1a | .635^**^ | .683^**^ | 1 |  |  |  |  |  |  |  |  |  |  |  |
| tortMainPathQ2a | .639^**^ | .703^**^ | .506^**^ | 1 |  |  |  |  |  |  |  |  |  |  |
| tortMainPathQ3a | .421^**^ | .480^**^ | .284^**^ | .278^**^ | 1 |  |  |  |  |  |  |  |  |  |
| tortMainPathQ4a | .507^**^ | .491^**^ | .346^**^ | .375^**^ | .318^**^ | 1 |  |  |  |  |  |  |  |  |
| tortQ1a | .635^**^ | .711^**^ | .742^**^ | .495^**^ | .361^**^ | .388^**^ | 1 |  |  |  |  |  |  |  |
| tortQ2a | .636^**^ | .757^**^ | .486^**^ | .740^**^ | .366^**^ | .349^**^ | .544^**^ | 1 |  |  |  |  |  |  |
| tortQ3a | .491^**^ | .524^**^ | .299^**^ | .378^**^ | .717^**^ | .302^**^ | .418^**^ | .431^**^ | 1 |  |  |  |  |  |
| tortQ4a | .566^**^ | .576^**^ | .388^**^ | .400^**^ | .394^**^ | .639^**^ | .463^**^ | .426^**^ | .365^**^ | 1 |  |  |  |  |
| tortQ1G1a | .644^**^ | .778^**^ | .869^**^ | .502^**^ | .328^**^ | .362^**^ | .841^**^ | .536^**^ | .373^**^ | .452^**^ | 1 |  |  |  |
| tortQ2G1a | .660^**^ | .805^**^ | .507^**^ | .879^**^ | .317^**^ | .382^**^ | .533^**^ | .848^**^ | .405^**^ | .431^**^ | .537^**^ | 1 |  |  |
| tortQ3G1a | .482^**^ | .530^**^ | .314^**^ | .331^**^ | .810^**^ | .293^**^ | .399^**^ | .409^**^ | .852^**^ | .347^**^ | .368^**^ | .345^**^ | 1 |  |
| tortQ4G1a | .567^**^ | .569^**^ | .390^**^ | .414^**^ | .384^**^ | .809^**^ | .448^**^ | .410^**^ | .367^**^ | .790^**^ | .425^**^ | .435^**^ | .358^**^ | 1 |

*Note.* torta= arteriolar tortuosity; Q1=quadrant 1; Q2= quadrant 2; Q3=quadrant 3; Q4=quadrant 4. G1=generation 1. Spearman rank correlation coefficients

* = *p* < .05; ** = *p* < .01

Supplementary Table S3b. Mean venular tortuosity correlations in the Lothian Birth Cohort 1936

|  | tortv | Tort  ImageG1v | Tort  MainPath  Q1v | Tort  MainPath  Q2v | Tort  MainPath  Q3v | Tort  MainPath  Q4v | Tort  Q1v | Tort  Q2v | Tort  Q3v | Tort  Q4v | Tort  Q1G1v | Tort  Q2G1v | Tort  Q3G1v | Tort  Q4G1v |
| --- | --- | --- | --- | --- | --- | --- | --- | --- | --- | --- | --- | --- | --- | --- |
| tortv | 1 |  |  |  |  |  |  |  |  |  |  |  |  |  |
| tortImageG1v | .450^**^ | 1 |  |  |  |  |  |  |  |  |  |  |  |  |
| tortMainPathQ1v | .239^**^ | .459^**^ | 1 |  |  |  |  |  |  |  |  |  |  |  |
| tortMainPathQ2v | .250^**^ | .504^**^ | .142^**^ | 1 |  |  |  |  |  |  |  |  |  |  |
| tortMainPathQ3v | .374^**^ | .497^**^ | .154^**^ | .231^**^ | 1 |  |  |  |  |  |  |  |  |  |
| tortMainPathQ4v | .358^**^ | .449^**^ | .186^**^ | .163^**^ | .151^**^ | 1 |  |  |  |  |  |  |  |  |
| tortQ1v | .341^**^ | .498^**^ | .516^**^ | .139^**^ | .227^**^ | .281^**^ | 1 |  |  |  |  |  |  |  |
| tortQ2v | .289^**^ | .539^**^ | .168^**^ | .537^**^ | .237^**^ | .217^**^ | .266^**^ | 1 |  |  |  |  |  |  |
| tortQ3v | .375^**^ | .474^**^ | .089^*^ | .229^**^ | .664^**^ | .245^**^ | .193^**^ | .284^**^ | 1 |  |  |  |  |  |
| tortQ4v | .435^**^ | .506^**^ | .213^**^ | .154^**^ | .238^**^ | .588^**^ | .249^**^ | .213^**^ | .347^**^ | 1 |  |  |  |  |
| tortQ1G1v | .235^**^ | .604^**^ | .783^**^ | .155^**^ | .206^**^ | .228^**^ | .615^**^ | .194^**^ | .167^**^ | .234^**^ | 1 |  |  |  |
| tortQ2G1v | .281^**^ | .646^**^ | .188^**^ | .787^**^ | .236^**^ | .199^**^ | .173^**^ | .665^**^ | .210^**^ | .222^**^ | .175^**^ | 1 |  |  |
| tortQ3G1v | .384^**^ | .535^**^ | .072 | .246^**^ | .849^**^ | .167^**^ | .212^**^ | .311^**^ | .797^**^ | .264^**^ | .146^**^ | .248^**^ | 1 |  |
| tortQ4G1v | .370^**^ | .508^**^ | .169^**^ | .143^**^ | .159^**^ | .770^**^ | .231^**^ | .177^**^ | .245^**^ | .737^**^ | .211^**^ | .174^**^ | .183^**^ | 1 |

*Note.* tortv = venular tortuosity; Q1=quadrant 1; Q2= quadrant 2; Q3=quadrant 3; Q4=quadrant 4. G1=generation 1. Spearman rank correlation coefficients

* = *p* < .05; ** = *p* < .01

Supplementary Table S4. Principal components analysis of the tortuosity measurements in the Lothian Birth Cohort 1396.

|  | First Unrotated Principal Component | |
| --- | --- | --- |
|  | Arteriolar | Venular |
| tortQ1 | .827 | .696 |
| tortQ2 | .750 | .574 |
| tortQ3 | .722 | .625 |
| tortQ4 | .743 | .583 |
| tortMainPathQ1 | .733 | .587 |
| tortMainPathQ2 | .723 | .653 |
| tortMainPathQ3 | .544 | .646 |
| tortMainPathQ4 | .605 | .607 |
| tortQ1G1 | .771 | .599 |
| tortQ2G1 | .729 | .611 |
| tortQ3G1 | .652 | .636 |
| tortQ4G1 | .765 | .654 |

Supplementary Table S5a. Correlations between measured arteriolar tortuosity variables and PCA derived scores in the Lothian Birth Cohort 1936

|  | torta | tortImageG1a | **tortQ1-Q4a** | **tortMainPath**  **Q1-Q4a** | **tortQ1G1-Q4G1a** |
| --- | --- | --- | --- | --- | --- |
| torta | 1 |  |  |  |  |
| TortImageG1a | .655** | 1 |  |  |  |
| **tortQ1-Q4a** | .720** | .850** | 1 |  |  |
| **tortMainPath Q1-Q4a** | .652** | .848** | .805** | 1 |  |
| **tortQ1G1-Q4G1a** | .698** | .931** | .904** | .885** | 1 |

*Note.* Bolded text=first unrotated factor scores.

* = p < .05; ** = p < .01.

Supplementary Table S5b. Correlations between measured venular tortuosity variables and PCA derived scores in the Lothian Birth Cohort 1936

|  | tortv | tortImageG1v | **tortQ1-Q4v** | **tortMainPath**  **Q1-Q4v** | **tortQ1G1-Q4G1v** |
| --- | --- | --- | --- | --- | --- |
| tortv | 1 |  |  |  |  |
| TortImageG1v | .538** | 1 |  |  |  |
| **tortQ1-Q4v** | .579** | .697** | 1 |  |  |
| **tortMainPath Q1-Q4v** | .498** | .787** | .603** | 1 |  |
| **tortQ1G1-Q4G1v** | .498** | .921** | .660** | .850** | 1 |

*Note.* Bolded text=first unrotated factor scores.

* = p < .05; ** = p < .01.

Supplementary Table S6a. Correlations between measurements calculated using Hermite and Spline methods of width estimations in the Lothian Birth Cohort 1936

|  | Hermite | | | | | | | |
| --- | --- | --- | --- | --- | --- | --- | --- | --- |
| Spline | BCa | BCv | AFa | AFv | JEa | JEv | LDRa | LDRv |
| BCa | -.050 |  |  |  |  |  |  |  |
| BCv | -.006 | .072 |  |  |  |  |  |  |
| AFa | -.077 | .004 | .136^**^ |  |  |  |  |  |
| AFv | .004 | .014 | -.038 | -.007 |  |  |  |  |
| JEa | -.008 | -.023 | .027 | -.023 | .012 |  |  |  |
| JEv | -.007 | -.100^*^ | -.053 | .088^*^ | -.003 | .094^*^ |  |  |
| LDRa | .169^**^ | -.011 | .068 | .080 | -.174^**^ | -.016 | .748^**^ |  |
| LDRv | .007 | .150^**^ | .084 | -.006 | -.012 | -.142^**^ | .037 | .830^**^ |

*Note.* BCa = branching coefficient arteriolar; BCv = branching coefficient venular; AFa = asymmetry factor arteriolar; AFv = asymmetry factor venular; JEa = junctional exponent arteriolar; JEv = junctional exponent venular; * = *p* < .05; ** = *p* < .01.

Supplementary Table S6b. Correlations between measurements calculated using Hermite and Spline methods of width estimations in the Lothian Birth Cohort 1936

|  | Hermite | | | | | | | |
| --- | --- | --- | --- | --- | --- | --- | --- | --- |
| Spline | GradQ1a | GradQ2a | GradQ3a | GradQ4a | GradQ1v | GradQ2v | GradQ3v | GradQ4v |
| GradQ1a | .140^**^ |  |  |  |  |  |  |  |
| GradQ2a | .114^*^ | .072 |  |  |  |  |  |  |
| GradQ3a | .100^*^ | -.006 | .021 |  |  |  |  |  |
| GradQ4a | .021 | .008 | .063 | .003 |  |  |  |  |
| GradQ1v | .090^*^ | .039 | -.021 | .091^*^ | .214^**^ |  |  |  |
| GradQ2v | .112^*^ | .021 | -.027 | .003 | .039 | .201^**^ |  |  |
| GradQ3v | .049 | .053 | .005 | -.007 | .047 | -.067 | .146^**^ |  |
| GradQ4v | -.009 | -.029 | .118^**^ | -.070 | .058 | -.041 | .066 | .140^**^ |

*Note.* GradQ1a = arteriolar width gradient in quadrant 1; GradQ2a = arteriolar width gradient in quadrant 2; GradQ3a = arteriolar width gradient in quadrant 3; GradQ4a = arteriolar width gradient in quadrant 4; GradQ1v = venular width gradient in quadrant 1; GradQ2v = venular width gradient in quadrant 2; GradQ3v = venular width gradient in quadrant 3; GradQ4v = venular width gradient in quadrant 4. * = *p* < .05; ** = *p* < .01.

|  | CRAE | CRVE | AVR | D0a | D0v | *Grad*  *Q1a* | *Grad*  *Q2a* | *Grad*  *Q3a* | *Grad*  *Q4a* | *Grad*  *Q1v* | *Grad*  *Q2v* | *Grad*  *Q3v* | *Grad*  *Q4v* | BSTDa | BSTDv | *BCa* | *BCv* | *AFa* | *AFv* | *JEa* | *JEv* | *LDRa* | *LDRv* | TORTa | TORTv |
| --- | --- | --- | --- | --- | --- | --- | --- | --- | --- | --- | --- | --- | --- | --- | --- | --- | --- | --- | --- | --- | --- | --- | --- | --- | --- |
| CRAE | 1 |  |  |  |  |  |  |  |  |  |  |  |  |  |  |  |  |  |  |  |  |  |  |  |  |
| CRVE | **.447^**^** | 1 |  |  |  |  |  |  |  |  |  |  |  |  |  |  |  |  |  |  |  |  |  |  |  |
| AVR | **.450**** | **-.577**** | 1 |  |  |  |  |  |  |  |  |  |  |  |  |  |  |  |  |  |  |  |  |  |  |
| D0a | **.341^**^** | **.358^**^** | -.059 | 1 |  |  |  |  |  |  |  |  |  |  |  |  |  |  |  |  |  |  |  |  |  |
| D0v | **.150^**^** | **.409^**^** | **-.276**** | **.601^**^** | 1 |  |  |  |  |  |  |  |  |  |  |  |  |  |  |  |  |  |  |  |  |
| *GradQ1a* | **-.197^**^** | **-.126^**^** | -.053 | **-.098^*^** | -.038 | 1 |  |  |  |  |  |  |  |  |  |  |  |  |  |  |  |  |  |  |  |
| *GradQ2a* | **-.099^*^** | **-.117^**^** | .034 | -.071 | **-.106^*^** | **.118^*^** | 1 |  |  |  |  |  |  |  |  |  |  |  |  |  |  |  |  |  |  |
| *GradQ3a* | **-.130^**^** | -.030 | **-.092*** | -.009 | **-.104^*^** | .002 | **-.098^*^** | 1 |  |  |  |  |  |  |  |  |  |  |  |  |  |  |  |  |  |
| *GradQ4a* | **-.140^**^** | **-.146^**^** | .018 | **-.113^**^** | **-.085^*^** | .040 | .062 | .083 | 1 |  |  |  |  |  |  |  |  |  |  |  |  |  |  |  |  |
| *GradQ1v* | -.015 | -.068 | .042 | -.081 | -.034 | **.182^**^** | -.018 | -.086 | .038 | 1 |  |  |  |  |  |  |  |  |  |  |  |  |  |  |  |
| *GradQ2v* | -.020 | .020 | -.041 | .030 | .027 | **.093^*^** | **.099^*^** | -.029 | .037 | .011 | 1 |  |  |  |  |  |  |  |  |  |  |  |  |  |  |
| *GradQ3v* | -.057 | -.056 | -.007 | -.071 | **-.109^*^** | .058 | **-.096^*^** | **.148^**^** | .089 | .002 | -.007 | 1 |  |  |  |  |  |  |  |  |  |  |  |  |  |
| *GradQ4v* | **-.114^**^** | **-.117^**^** | .007 | **-.100^*^** | **-.157^**^** | .028 | -.087 | .070 | .056 | **.159^**^** | -.029 | .086 | 1 |  |  |  |  |  |  |  |  |  |  |  |  |
| BSTDa | **.424^**^** | .046 | **.329**** | **-.195^**^** | **-.097^*^** | -.064 | -.064 | .006 | -.037 | .041 | .001 | -.034 | -.020 | 1 |  |  |  |  |  |  |  |  |  |  |  |
| BSTDv | **.147^**^** | -.045 | **.171**** | **-.108^**^** | **-.234^**^** | -.040 | -.043 | .029 | .064 | -.089^*^ | -.080 | .041 | -.008 | **.142^**^** | 1 |  |  |  |  |  |  |  |  |  |  |
| *BCa* | **-.099^*^** | .067 | **-.165**** | **.134^**^** | **.112^**^** | -.067 | .077 | **.127^**^** | -.011 | -.021 | -.027 | .021 | .011 | **-.151^**^** | -.077 | 1 |  |  |  |  |  |  |  |  |  |
| *BCv* | .003 | .020 | -.024 | -.033 | -.033 | .013 | -.028 | -.035 | -.056 | -.021 | -.027 | .031 | .024 | .027 | -.077 | **.090^*^** | 1 |  |  |  |  |  |  |  |  |
| *AFa* | **-.157^**^** | **-.198^**^** | .049 | -.110^*^ | -.049 | .062 | .085 | .034 | .041 | .077 | .031 | .023 | **.093^*^** | .073 | **-.097^*^** | **-.104^*^** | -.044 | 1 |  |  |  |  |  |  |  |
| *AFv* | **-.146^**^** | **-.139^**^** | -.003 | -.082 | -.061 | .072 | **.120^**^** | .008 | .051 | .033 | .044 | -.069 | .059 | **.091^*^** | **-.127^**^** | **-.112^*^** | **-.093^*^** | **.286^**^** | 1 |  |  |  |  |  |  |
| *JEa* | **.156^**^** | -.034 | **.182**** | **-.118^**^** | **-.100^*^** | .040 | **-.100^*^** | **-.109^*^** | .002 | .040 | .007 | .020 | -.022 | **.174^**^** | .070 | **-.939^**^** | -.057 | .065 | .074 | 1 |  |  |  |  |  |
| *JEv* | .020 | .057 | -.021 | -.012 | .013 | -.033 | .007 | .038 | .005 | -.009 | -.060 | -.052 | -.037 | -.018 | .055 | -.031 | **-.900^**^** | **-.092^*^** | -.043 | -.008 | 1 |  |  |  |  |
| *LDRa* | .023 | .081 | -.067 | **.087^*^** | .065 | .048 | .002 | .046 | -.006 | .032 | **-.093^*^** | -.055 | .016 | .040 | .033 | **.173^**^** | .016 | **-.112^**^** | -.010 | **-.152^**^** | .010 | 1 |  |  |  |
| *LDRv* | .013 | -.047 | .046 | .080 | .027 | .034 | -.007 | .033 | -.039 | -.004 | -.033 | -.035 | .030 | .022 | .071 | .028 | **.089^*^** | -.003 | -.045 | -.016 | **-.112^**^** | **.122^**^** | 1 |  |  |
| *TORTa* | .042 | .046 | .002 | -.063 | -.067 | .028 | .019 | **-.090*** | -.013 | -.012 | .035 | -.019 | -.028 | **.081*** | -.051 | -.011 | .004 | -.039 | -.005 | .005 | .020 | .086 | .027 |  |  |
| *TORTv* | .070 | .065 | -.006 | -.030 | .017 | -.053 | -.037 | **-.088*** | -.006 | -.003 | -.034 | .015 | .023 | .043 | .023 | .026 | .065 | -.070 | **-.085*** | .033 | -.022 | .080 | .052 | **.155**** | 1 |

Supplementary Table S7a. Correlations between retinal width, fractal dimension, branching geometry and tortuosity in the Lothian Birth Cohort 1936 (widths calculated using the Hermite technique)

*Note.* CRAE=central retinal artery equivalent; CRVE=central vein retinal equivalent; AVR=arteriole-venular ratio; D0a= multifractal (capacity) dimension arteriolar; D0v=multifractal (capacity) dimension venular; GradQ1a=arteriolar width gradient in quadrant 1; GradQ2a=arteriolar width gradient in quadrant 2; GradQ3a=arteriolar width gradient in quadrant 3; GradQ4a=arteriolar width gradient in quadrant 4; GradQ1v=venular width gradient in quadrant 1; GradQ2v=venular width gradient in quadrant 2; GradQ3v=venular width gradient in quadrant 3; GradQ4v=venular width gradient in quadrant 4; BSTDa=standard deviation of arteriolar width in Zone B; BSTDv=standard deviation of venular width in Zone B; BCa=branching coefficient arteriolar; BCv=branching coefficient venular; AFa=asymmetry factor arteriolar; AFv=asymmetry factor venular; JEa=junctional exponent arteriolar; JEv=junctional exponent venular; LDRa=length diameter ratio arteriolar; length diameter ratio venular. TORTa=tortImageG1a; TORTv=tortImageG1v (tortuosity variables log transformed). Variables in italics calculated using Hermite technique. * = *p* < .05; ** = *p* < .01.

Supplementary Table S7b. Correlations between retinal width, fractal dimension, branching geometry and tortuosity correlations in the Lothian Birth Cohort 1936 (widths calculated using the Spline technique)

|  | CRAE | CRVE | AVR | D0a | D0v | *Grad*  *Q1a* | *Grad*  *Q2a* | *Grad*  *Q3a* | *Grad*  *Q4a* | *Grad*  *Q1v* | *Grad*  *Q2v* | *Grad*  *Q3v* | *Grad*  *Q4v* | BSTDa | BSTDv | *BCa* | *BCv* | *AFa* | *AFv* | *JEa* | *JEv* | *LDRa* | *LDRv* | *TORTa* | *TORTv* |
| --- | --- | --- | --- | --- | --- | --- | --- | --- | --- | --- | --- | --- | --- | --- | --- | --- | --- | --- | --- | --- | --- | --- | --- | --- | --- |
| CRAE | 1 |  |  |  |  |  |  |  |  |  |  |  |  |  |  |  |  |  |  |  |  |  |  |  |  |
| CRVE | **.447^**^** | 1 |  |  |  |  |  |  |  |  |  |  |  |  |  |  |  |  |  |  |  |  |  |  |  |
| AVR | **.450**** | **-.577**** | 1 |  |  |  |  |  |  |  |  |  |  |  |  |  |  |  |  |  |  |  |  |  |  |
| D0a | **.341^**^** | **.358^**^** | -.059 | 1 |  |  |  |  |  |  |  |  |  |  |  |  |  |  |  |  |  |  |  |  |  |
| D0v | **.150^**^** | **.409^**^** | **-.276**** | **.601^**^** | 1 |  |  |  |  |  |  |  |  |  |  |  |  |  |  |  |  |  |  |  |  |
| *GradQ1a* | **-.155**** | -.010 | **-.122**** | **-.103**** | .024 | 1 |  |  |  |  |  |  |  |  |  |  |  |  |  |  |  |  |  |  |  |
| *GradQ2a* | **-.139**** | .012 | **-.123**** | **-.121**** | -.009 | **.111*** | 1 |  |  |  |  |  |  |  |  |  |  |  |  |  |  |  |  |  |  |
| *GradQ3a* | **-.148**** | .006 | **-.136**** | .019 | .072 | .**148**** | .091 | 1 |  |  |  |  |  |  |  |  |  |  |  |  |  |  |  |  |  |
| *GradQ4a* | **-.172**** | **-.116**** | -.038 | **-.116**** | -.075 | .073 | **.145**** | **.091*** | 1 |  |  |  |  |  |  |  |  |  |  |  |  |  |  |  |  |
| *GradQ1v* | -.070 | **-.216**** | **.139**** | **-.195**** | **.094*** | **.264**** | **.150**** | .063 | .079 | 1 |  |  |  |  |  |  |  |  |  |  |  |  |  |  |  |
| *GradQ2v* | -.056 | .074 | .032 | **-.149**** | **-.096*** | **.111*** | **.234**** | .068 | **.108*** | .091 | 1 |  |  |  |  |  |  |  |  |  |  |  |  |  |  |
| *GradQ3v* | -.015 | .031 | -.033 | .012 | .031 | .054 | .044 | **.300**** | **.153**** | -.005 | .**099*** | 1 |  |  |  |  |  |  |  |  |  |  |  |  |  |
| *GradQ4v* | -.029 | -.077 | .034 | -.026 | -.002 | .044 | **.090*** | **.163**** | **.279**** | .062 | .061 | .**220**** | 1 |  |  |  |  |  |  |  |  |  |  |  |  |
| BSTDa | **.424**** | .046 | **.329**** | **-.195**** | **-.097*** | -.046 | .006 | -.023 | .078 | **.100*** | .021 | .017 | .013 | 1 |  |  |  |  |  |  |  |  |  |  |  |
| BSTDv | **.147**** | -.045 | **.171**** | **-.108**** | **-.234**** | .004 | .040 | .040 | -.046 | -.038 | -.031 | -.001 | **.088*** | **.142**** | 1 |  |  |  |  |  |  |  |  |  |  |
| *BCa* | -.054 | -.022 | -.025 | -.063 | .028 | .075 | .078 | .036 | **.107*** | .074 | .083 | .070 | .078 | -.019 | .057 | 1 |  |  |  |  |  |  |  |  |  |
| *BCv* | .008 | -.034 | .049 | .014 | .028 | -.015 | -.030 | .013 | .047 | -.010 | .074 | .032 | .030 | -.002 | -.010 | .038 | 1 |  |  |  |  |  |  |  |  |
| *AFa* | -.042 | -.012 | -.019 | .016 | -.023 | .048 | -.076 | .041 | -.045 | .046 | .039 | -.002 | -.041 | -.024 | -.019 | .082 | .056 | 1 |  |  |  |  |  |  |  |
| *AFv* | **-.086*** | -.015 | -.046 | **-.101*** | .021 | .049 | -.040 | .076 | .040 | -.024 | -.024 | .021 | -.004 | .009 | -.035 | .002 | **.196**** | .019 | 1 |  |  |  |  |  |  |
| *JEa* | .050 | .077 | -.029 | .044 | -.025 | **-.117*** | -.042 | -.036 | -.063 | -.079 | -.044 | -.085 | -.076 | .049 | -.043 | **-.791**** | -.039 | -.016 | -.030 | 1 |  |  |  |  |  |
| *JEv* | .027 | .046 | -.030 | .006 | -.012 | .058 | .005 | .006 | -.041 | .016 | -.062 | -.006 | -.042 | .033 | -.019 | -.011 | **-.785**** | -.045 | -.020 | .025 | 1 |  |  |  |  |
| *LDRa* | **-.212**** | -.064 | **-.136**** | .046 | **.094*** | .046 | .081 | **.171**** | **.178**** | **.092*** | .057 | **.102*** | .061 | .004 | -.044 | **.123**** | -.089 | .034 | -.005 | -.083 | .020 | 1 |  |  |  |
| *LDRv* | -.047 | **-.125**** | .075 | .064 | .068 | .011 | .**135**** | .044 | **.135**** | .048 | .040 | **.123**** | .080 | .019 | -.041 | -.055 | .029 | .033 | .027 | .068 | -.069 | **.110*** | 1 |  |  |
| *TORTa* | .042 | .046 | .002 | -.063 | -.067 | -.060 | -.036 | .021 | .017 | .001 | -.002 | .068 | .002 | **.081*** | -.051 | -.010 | .040 | -.062 | .039 | .022 | -.002 | .029 | -.034 | 1 |  |
| *TORTv* | .070 | .065 | -.006 | -.030 | .017 | -.043 | -.025 | -.074 | **-.095*** | -.057 | **-.100*** | **-.118**** | -.033 | .042 | .023 | .053 | **.115**** | -.019 | .080 | -.064 | -.076 | -.030 | -.006 | .**155**** | 1 |

*Note.* CRAE=central retinal artery equivalent; CRVE=central vein retinal equivalent; AVR=arteriole-venular ratio; D0a= multifractal (capacity) dimension arteriolar; D0v=multifractal (capacity) dimension venular; GradQ1a=arteriolar width gradient in quadrant 1; GradQ2a=arteriolar width gradient in quadrant 2; GradQ3a=arteriolar width gradient in quadrant 3; GradQ4a=arteriolar width gradient in quadrant 4; GradQ1v=venular width gradient in quadrant 1; GradQ2v=venular width gradient in quadrant 2; GradQ3v=venular width gradient in quadrant 3; GradQ4v=venular width gradient in quadrant 4; BSTDa=standard deviation of arteriolar width in Zone B; BSTDv=standard deviation of venular width in Zone B; BCa=branching coefficient arteriolar; BCv=branching coefficient venular; AFa=asymmetry factor arteriolar; AFv=asymmetry factor venular; JEa=junctional exponent arteriolar; JEv=junctional exponent venular; LDRa=length diameter ratio arteriolar; length diameter ratio venular. TORTa=tortImageG1a; TORTv=tortImageG1v (tortuosity variables log transformed). Variables in italics calculated using Spline technique. * = *p* < .05; ** = *p* < .01

Supplementary Table S8. Principal components analysis of the gradient measurements in the Lothian Birth Cohort 1396.

|  | First Unrotated Principal Component | |
| --- | --- | --- |
|  | Arteriolar | Venular |
| Gradient Q1 | .579 | .290 |
| Gradient Q2 | .605 | .481 |
| Gradient Q3 | .574 | .696 |
| Gradient Q4 | .539 | .698 |

Supplementary Table S9. Correlations between retinal variables selected though data reduction in the Lothian Birth Cohort 1936

|  | CRAE | CRVE | BSTDa | BSTDv | FDa | FDv | BCa | BCv | AFa | AFv | LDRa | LDRv | Gradient a | Gradient v | TORTa | TORTv |
| --- | --- | --- | --- | --- | --- | --- | --- | --- | --- | --- | --- | --- | --- | --- | --- | --- |
| CRAE | 1 |  |  |  |  |  |  |  |  |  |  |  |  |  |  |  |
| CRVE | **.447**** | 1 |  |  |  |  |  |  |  |  |  |  |  |  |  |  |
| BSTDa | **.424**** | .046 | 1 |  |  |  |  |  |  |  |  |  |  |  |  |  |
| BSTDv | **.147**** | -.045 | **.142**** | 1 |  |  |  |  |  |  |  |  |  |  |  |  |
| FDa | **.341**** | **.358**** | **-.195**** | **-.108**** | 1 |  |  |  |  |  |  |  |  |  |  |  |
| FDv | **.150**** | **.409**** | **-.097*** | **-.234**** | **.601**** | 1 |  |  |  |  |  |  |  |  |  |  |
| BCa | **-.099*** | .067 | **-.151**** | -.077 | **.134**** | **.112**** | 1 |  |  |  |  |  |  |  |  |  |
| BCv | .003 | .020 | .027 | -.077 | -.033 | -.033 | **.090*** | 1 |  |  |  |  |  |  |  |  |
| AFa | **-.157**** | **-.198**** | .073 | **-.097*** | **-.110*** | -.049 | **-.104*** | -.044 | 1 |  |  |  |  |  |  |  |
| AFv | **-.146**** | **-.139**** | **.091*** | **-.127**** | -.082 | -.061 | **-.112*** | **-.093*** | **.286**** | 1 |  |  |  |  |  |  |
| LDRa | .023 | .081 | .040 | .033 | **.087*** | .065 | **.173**** | .016 | **-.112**** | -.010 | 1 |  |  |  |  |  |
| LDRv | .013 | -.047 | .022 | .071 | .080 | .027 | .028 | **.089*** | -.003 | -.045 | **.122**** | 1 |  |  |  |  |
| Gradient a | **-.266**** | -.049 | .031 | .003 | **-.207**** | -.024 | -.031 | .051 | .097 | .031 | .067 | .083 | 1 |  |  |  |
| Gradient v | -.053 | -.087 | .057 | .008 | **-.112*** | -.045 | -.058 | -.015 | .036 | .035 | .108 | .072 | **.371**** | 1 |  |  |
| TORTa | .042 | .046 | **.081*** | -.051 | -.063 | -.067 | -.011 | .004 | -.039 | -.005 | .082 | .027 | -.010 | .025 | 1 |  |
| TORTv | .070 | .065 | .042 | .023 | -.030 | .017 | -.028 | .065 | -.070 | **-.085*** | .063 | .052 | -.083 | **-.125*** | .**155**** | 1 |

*Note.* CRAE=central retinal artery equivalent; CRVE=central vein retinal equivalent; BSTDa=standard deviation of arteriolar width in Zone B; BSTDv=standard deviation of venular width in Zone B; FDa= multifractal (capacity) dimension arteriolar; FDv=multifractal (capacity) dimension venular; BCa=branching coefficient arteriolar; BCv=branching coefficient venular; AFa=asymmetry factor arteriolar; AFv=asymmetry factor venular; LDRa=length diameter ratio arteriolar; LDRv=length diameter ratio venular; Gradient a=FUPC arteriolar width gradient; Gradient v=FUPC venular width gradient; TORTa=tortImageG1a, arteriolar tortuosity of generation 1 vessels; TORTv=tortImageG1v, venular tortuosity of generation 1 vessels.

* = *p* < .05; ** = *p* < .01

Supplementary Table S10: Principal components analysis of the vessel width and fractal measurements in the Lothian Birth Cohort 1396.

|  | First unrotated  principal component |
| --- | --- |
| CRAE | .621 |
| CRVE | .750 |
| FDa | .803 |
| FDv | .757 |

*Note.* CRAE=central retinal artery equivalent; CRVE=central vein retinal equivalent; FDa= multifractal (capacity) dimension arteriolar; FDv=multifractal (capacity) dimension venular


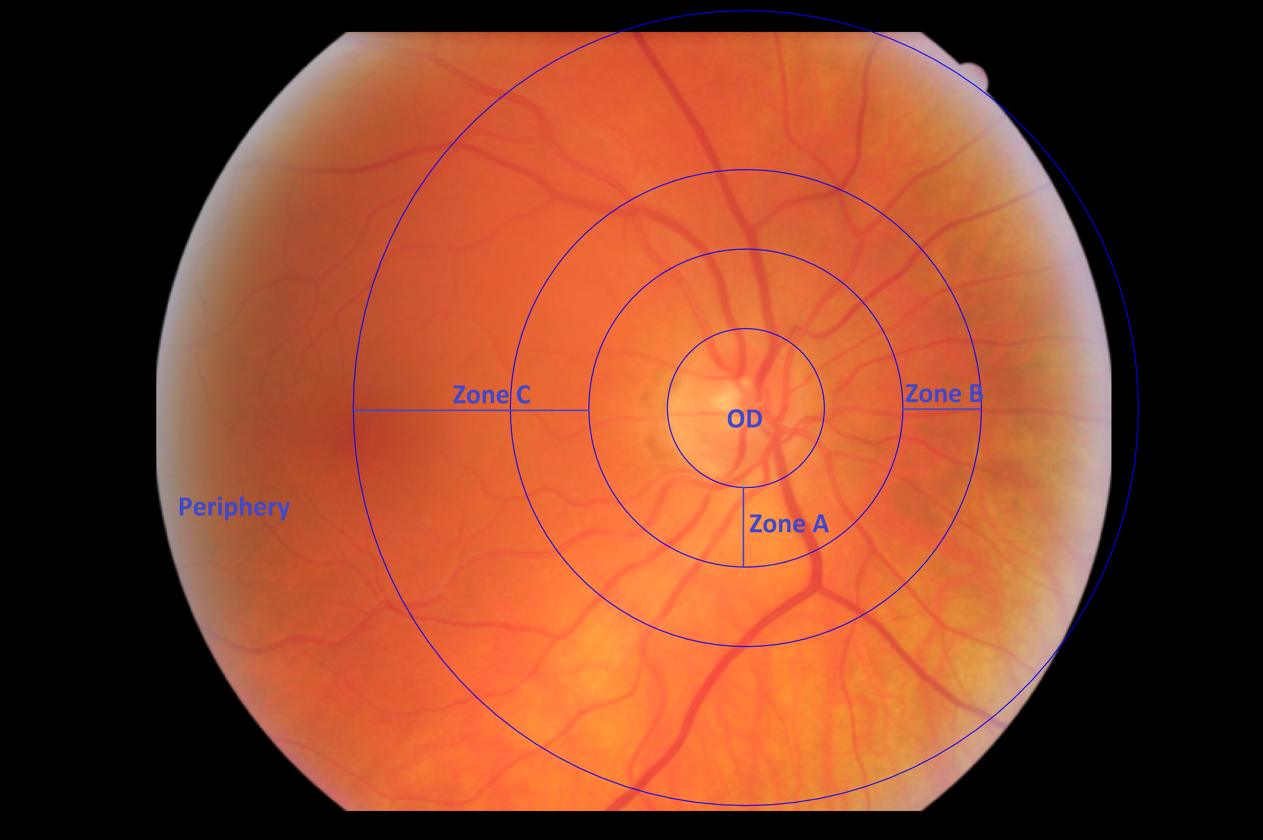


Supplementary Figure S1. Retinal image zones.

Image is divided into concentric regions centred on the optic disc centre. OD region: area within 0.5 optic disc diameters (ODD) from the image centre; Zone A: Annulus between 0.5 and 1.0 ODD from OD centre; Zone B: Annulus between 1.0 and 1.5 ODD; Zone C: Annulus between 1.0 and 2.5 ODD.

286

Retinal Measurements

143 left eye measurements

143 right eye measurements

143

mean retinal measurements

100 tortuosity

*PCA used to explore data*

6 fractal dimension

*All strongly correlated*

*(r >.98)*

16 gradient & branching geometry

(Calculated twice by Hermite/Spline methods)

*Inter-retinal correlations used to select appropriate method for each variable*

tortImageG1a

tortmageG1v

D0a

D0v

Hermite Spline

BCa BCv GradQ1a GradQ1v

JEa JEv GradQ2a GradQ2v

AFa AFv GradQ3a GradQ3v

LDRa LDRv GradQ4a GradQ4v

5 vessel

width:

CRAE, CRVE

AVR

BSTDa, BSTDv

**AVR excluded**

*As a ratio of CRAE and CRVE, information provided by CRAE and CRVE*

Gradient

measures

*PCA to reduce quadrants*

BC and JE strongly correlated (r > -.9)

**JE excluded**

BCa, BCv, AFa, AFV, LDRa, LDRv

Branching geometry

(BC, JE, AF, LDR)

FUPC Grad a

FUPC Grad v

CRAE, CRVE,

BSTDa, BSTDv

Supplementary Figure S2. Flow chart illustrating data reduction

PCA=principal components analysis; tortImageG1a/v=tortuosity of generation 1 arterioles/venules; D0a/v= multifractal (capacity) dimension arteriolar/venular; CRAE=central retinal artery equivalent; CRVE=central vein retinal equivalent; AVR=arteriole-venular ratio; BSTDa/v=standard deviation of arteriolar/venular width in Zone B; BCa=branching coefficient arteriolar; AFa=asymmetry factor arteriolar; LDRa=length diameter ratio arteriolar; JE=junctional exponent; GradQ1-Q4a/v=width gradient in quadrant 1-4 arteriolar/venular. FUPC Grad a=first unrotated principal component arteriolar width gradient; FUPC Grad v=first unrotated principal component venular width gradient


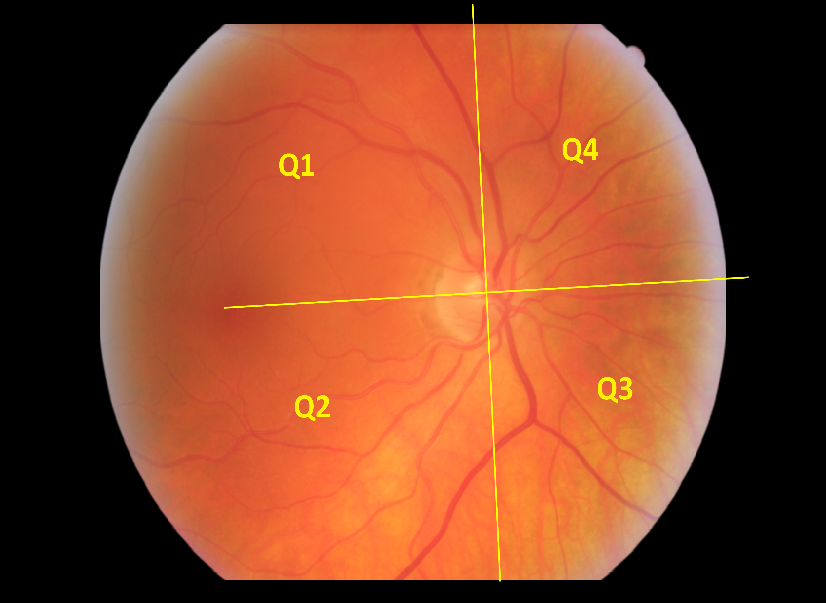


Supplementary Figure S3. Image quadrants.

Quadrants numbered according to the following convention: Q1=Supratemporal; Q2=Inferotemporal; Q3=Inferonasal; Q4=Supranasal.


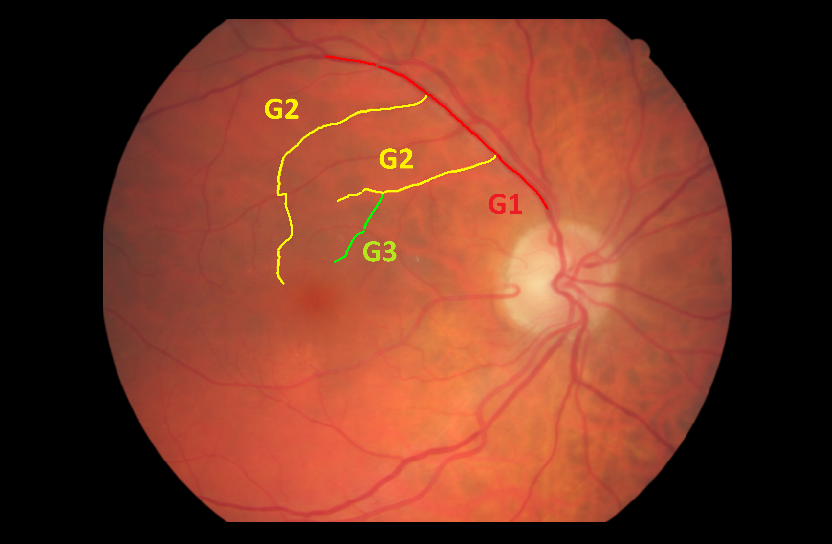


Supplementary Figure S4. Tortuosity Q1G1-Q4G1.

The main path of each tree is labelled as the “Generation 1” (G1) vessel


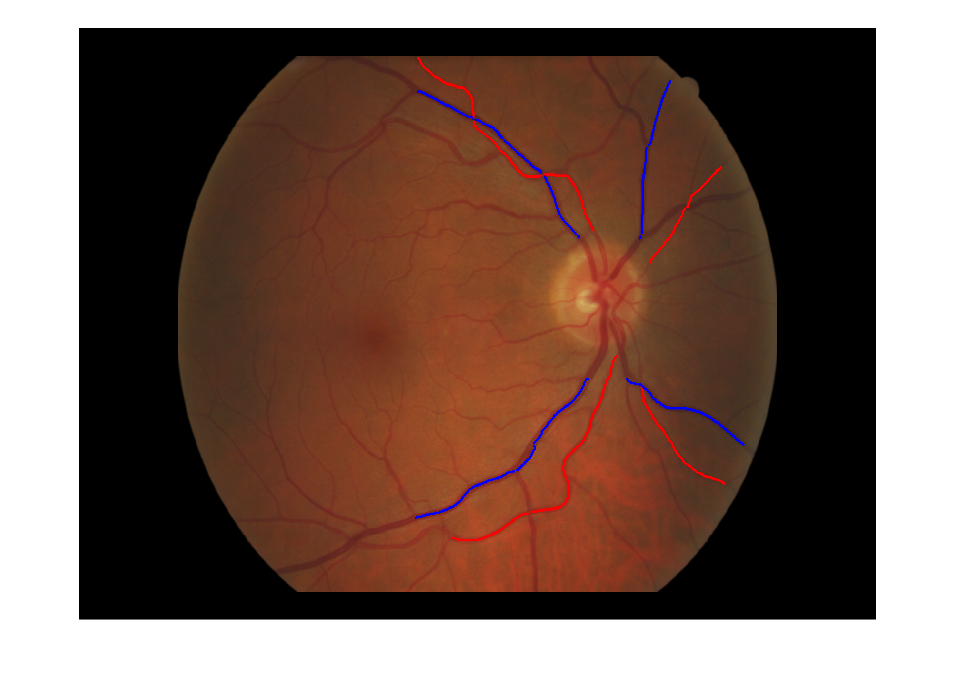


Supplementary Figure S5. Tortuosity Main Path Q1-Q4.

In each quadrant, one main arteriolar (red) path and one main venular path (blue) is identified. Main path vessels are also used in the calculation of the width gradient variables.

**tortQ1-Q4a**

**tortMainPathQ1-Q4a**


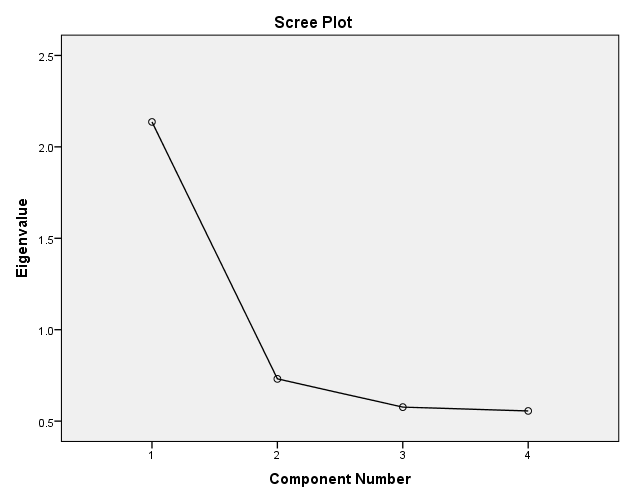


**tortQ1G1-Q4G1a**

**tortQ1-Q4v**


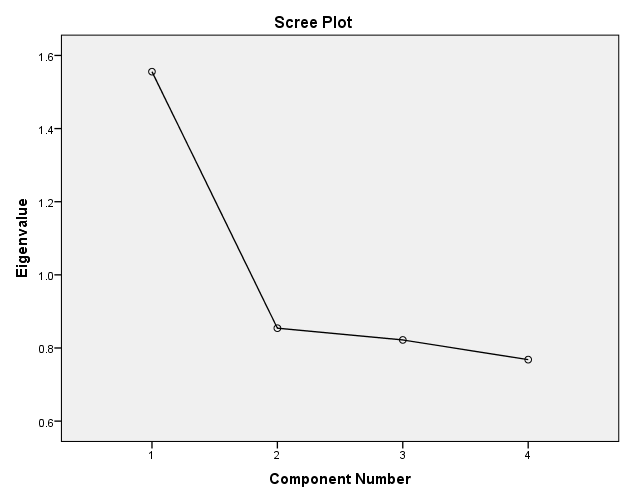


**tortMainPathQ1-Q4v**

**tortQ1G1-Q4G1a**

Supplementary Figure S6.

Scree plots for the principal component factor analysis based on quadrant tortuosity measurements. In each case, the eigenvalues of the first component and the decreasing eigenvalues of the remaining components indicate a single factor underlying the tortuosity across retinal quadrants.


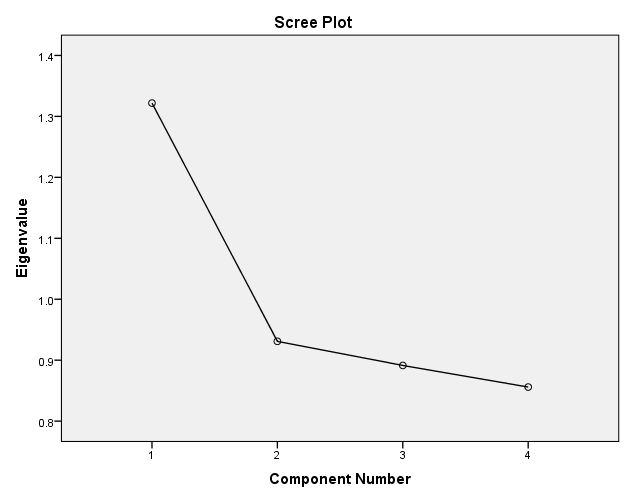

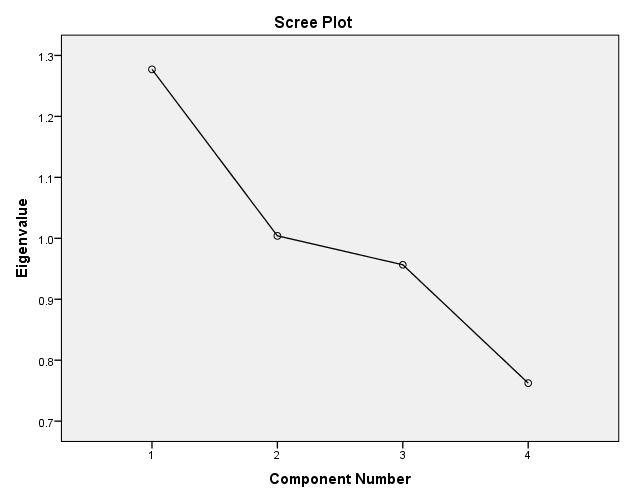


**Arteriolar width gradient**

**Venular**

**width gradient**

Supplementary Figure S7.

Scree plots for the principal component factor analysis based on vessel width gradient measurements.

**c**

**b**

**a**


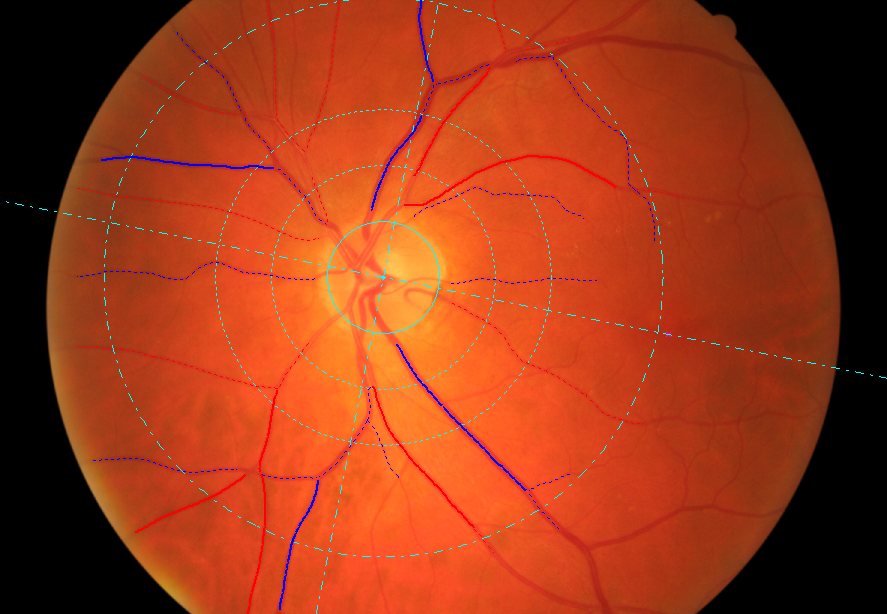


**Zone C**

**Zone B**

**OD**


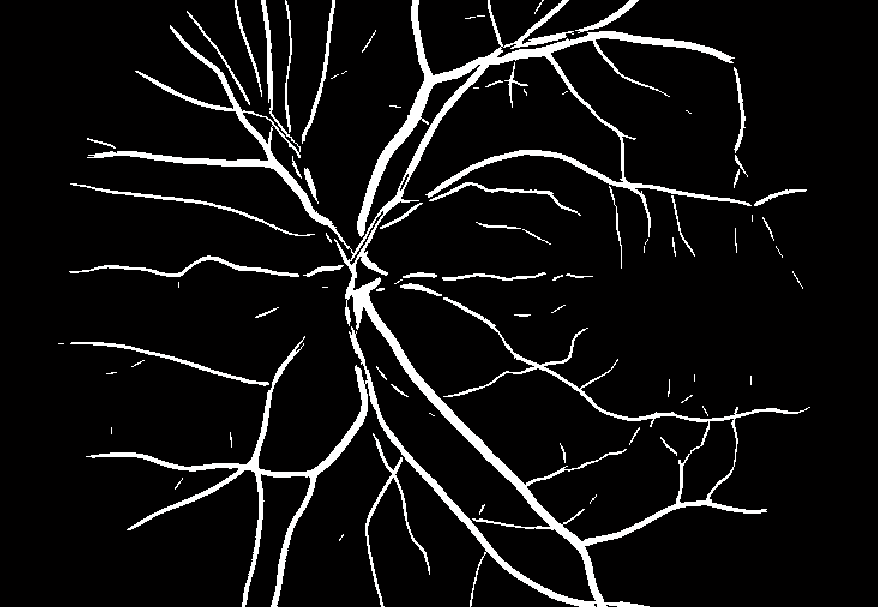

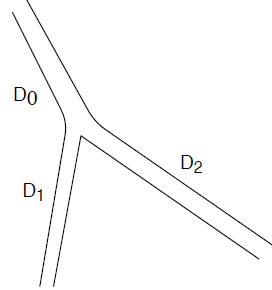

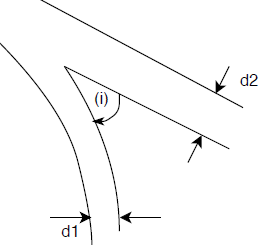


**d**

**e**

**f**

**e**


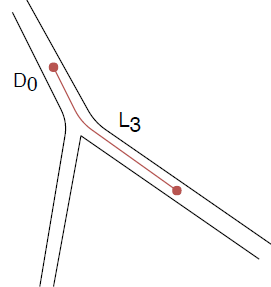


**a-c**


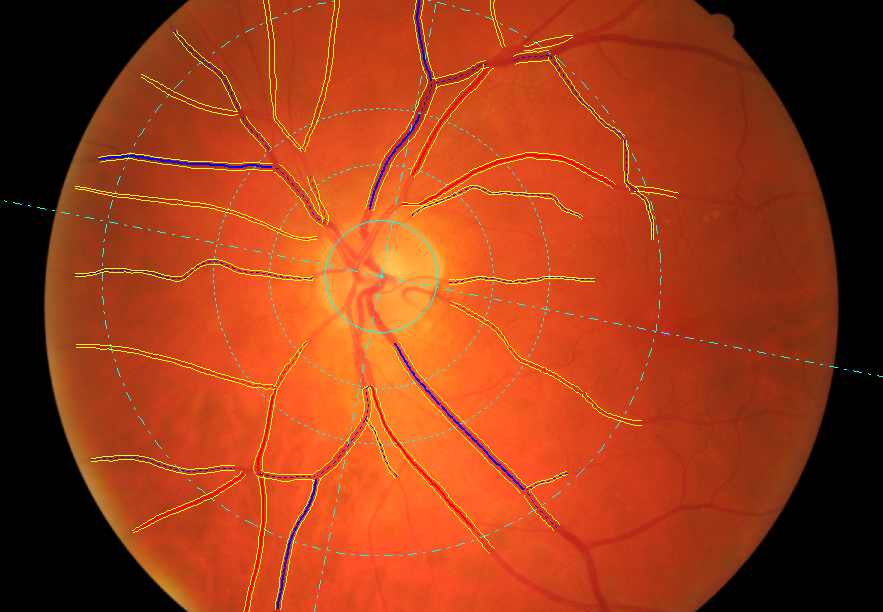


**g**


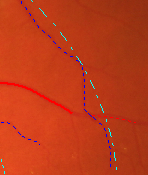


**f**


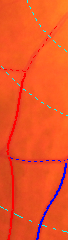


D_2_

D_1_

Supplementary Figure S8. Retinal measurements derived using VAMPIRE

*Note.* OD=optic disc; a=length diameter ratio (the length from the midpoint of the first branch to the midpoint of the second branch [L_3_] divided by the diameter of the first branch [D_0_]); b=branching coefficient (calculated using the following formula, where D_0_ is the parent diameter and D_1_ and D_2_ the daughter diameters [(D_1_^2^ + D_2_^2^)/D_0_^2^]); c=asymmetry factor (ratio of the squares of the 2 branching vessel widths [D_1_^2^/D_2_^2^]). (i) indicates the branching angle; d=binary map used to calculate fractal dimension; e=vessel width boundaries for width estimation (CRAE, CRVE, BSTDa, BSTDv); f=tortuosity; g=vessel width gradient (In each quadrant, one main arterial path and one main venular paths are identified. The path lengths are accounted for in the calculation of the width gradient measurements).


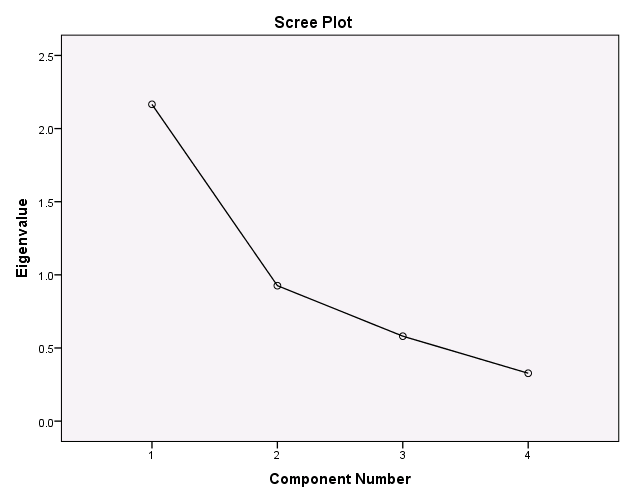


Supplementary Figure S9.

Scree plots for the principal component factor analysis based on vessel width and fractal dimension measurements.

High

BP

Systolic BP

Diastolic

BP

Smoking

Diabetes

Blood HbA1C

Cholesterol

High cholesterol

0.401

-0.143

-0.216

0.055

0.253

0.222

-0.561

0.642

0.108

0.561

0.649

0.341

-0.194

χ²/df=23/15 (p=0.07)

Max MI=5

CFI=0.971

TLI=0.947

RMSEA=0.031

Supplementary Figure S10. Diagram of measurement model for VRF in LBC1936.

Rectangles represent measured variables; ellipses, latent variables. Single-headed arrows are hypothesised causal pathways; double-headed arrows are correlations. Model fit indices are shown: a nonsignificant χ^2^ indicates a well-fitting model; Max MI=maximum modification index; CFI=comparative fit index; TLI= Tucker-Lewis index; RMSEA=root mean square error of approximation; VRF=vascular risk factor; HbA1c=haemoglobin A1c; All variables adjusted for age and sex. Standardised loadings and residual correlations are shown

WMH % ICV

Fazekas Peri.

Fazekas Deep

0.949

0.782

0.715

Lacunes

Fazekas Peri.

Fazekas Deep

PVS

Micro

bleeds

0.744

0.739

0.435

0.057

0.204

CFI=0.99

TLI=0.99

RMSEA=0.067

CFI=1.00

TLI=1.020

RMSEA=0.00

Supplementary Figure S11. Diagram of measurement model for WMH and SVD latent variable constructs in LBC1936.

Rectangles represent measured variables; ellipses, latent variables. Single-headed arrows are hypothesised causal pathways. Model fit indices are shown: CFI=comparative fit index; TLI= Tucker-Lewis index; RMSEA=root mean square error of approximation. WMH=white matter hyperintensities; ICV= intracranial volume; Peri.= Periventricular; SVD=small vessel disease; PVS = basal ganglia perivascular spaces. Standardised loadings shown. Each variable adjusted for sex and age.

FDa

FDv

CRAE

CRVE

0.648

0.798

0.664

0.583

-0.822

CFI=0.99

TLI=0.93

RMSEA=0.080

Supplementary Figure S12. Diagram of retinal measurement models in LBC1936.

Rectangles represent measured variables; ellipses, latent variables. Single-headed arrows are hypothesised causal pathways; double-headed arrows are correlations. Model fit indices are shown: a nonsignificant χ^2^ indicates a well-fitting model; Max MI=maximum modification index; CFI=comparative fit index; TLI= Tucker-Lewis index; RMSEA=root mean square error of approximation; FDa=fractal dimension arteriolar; FDv=fractal dimension venular; CRAE=central retinal artery equivalent; CRVE=central retinal vein equivalent; All variables adjusted for age and sex. Standardised loadings and residual correlations are shown

**a b**

.178

.239

.081

-.189

.084

Age

Sex

FDa

WMH

FDa

WMH

VRF

WMH

WMH % BTV

.140

.179

.239

.080

-.193

.082

Age

Sex

FDa

WMH

FDa

WMH

VRF

WMH % ICV

.143

**c**

.192

.234

.089

-.174

.016

Age

Sex

FDa

FDa

VRF

WMH

Fazekas Peri.

Supplementary Figure S13. Structural models for FDa predicting measured brain imaging variables in the LBC1936

(a) Model of FDa predicting WMH % in BTV; (b) Model of FDa predicting WMH % in ICV; (c) Model of FDa predicting Fazekas Periventricular score. Standardised regression coefficients (parameter weights) are shown adjacent to each path. P-values corrected for False Discovery Rate. The rectangles to the left are the covariates in the model. Each of the covariates was examined for their contribution to VRF, FDa and brain imaging-derived parameter, and only significant (*p* < .05) contributions are shown. FDa = arteriolar fractal dimension; VRF = vascular risk factor; WMH % in ICV = white matter hyperintensities volume as a percentage of intracranial volume; WMH % BTV = white matter hyperintensities as a percentage of brain tissue volume; Fazekas Peri = Fazekas ratings in periventricular regions. Dashed lines represent non-significant associations.

**References**

1. Couper, D. J. et al. Reliability of retinal photography in the assessment of retinal microvascular characteristics: the Atherosclerosis Risk in Communities Study. *Am J Ophthalmol.* **133**(1), 78-88 (2002).
2. Ikram, M. K. et al. Retinal vessel diameters and risk of hypertension: the Rotterdam Study. *Hypertension.* **47**(2), 189-94 (2006).
3. Leung, H. et al. Computer‐assisted retinal vessel measurement in an older population: correlation between right and left eyes. *Clin. Exp. Ophthamol*. **31**(4), 326-30 (2003).
4. Wong, T. Y. et al. Computer-assisted measurement of retinal vessel diameters in the Beaver Dam Eye Study: methodology, correlation between eyes, and effect of refractive errors. *Ophthalmology.* **111**(6), 1183-90 (2004).
5. Doubal, F. N. et al. Fractal analysis of retinal vessels suggests that a distinct vasculopathy causes lacunar stroke. *Neurology.* **74**(14), 1102-7 (2010).
6. Taylor, A. M. et al. Retinal vascular fractal dimension, childhood IQ, and cognitive ability in old age: the Lothian birth cohort study 1936. *PloS One*. **10**(3):e0121119 (2015).
7. MacGillivray, T.J. et al. Suitability of UK Biobank retinal images for automatic analysis of morphometric properties of the vasculature. *PLoS One.* **10**(5):e0127914 (2015).
8. Baker, M. L. et al. Retinal microvascular signs, cognitive function, and dementia in older persons: the Cardiovascular Health Study. *Stroke.* **38**(7), 2041-7 (2007).
9. Baker, M. L. et al. Early age-related macular degeneration, cognitive function, and dementia: the Cardiovascular Health Study. *Arch. Ophthalmol.* **127**(5), 667-73 (2009).
10. Hubbard, L. D. et al. Methods for evaluation of retinal microvascular abnormalities associated with hypertension/sclerosis in the atherosclerosis risk in communities study1. *Ophthalmology.* **106**(12), 2269-80 (1999).
11. Klein, R. et al. Are retinal arteriolar abnormalities related to atherosclerosis?: The Atherosclerosis Risk in Communities Study. *Arterioscler. Thromb. Vasc. Biol.* **20**(6), 1644-50 (2000).
12. Wong, T. Y. et al. Retinal microvascular abnormalities and incident stroke: the Atherosclerosis Risk in Communities Study. *Lancet.* **358**(9288), 1134-40 (2001).
13. Wong, T. Y. et al. Retinal arteriolar narrowing and risk of coronary heart disease in men and women: the Atherosclerosis Risk in Communities Study. *JAMA*. **287**(9), 1153-9 (2002).
14. Hemminki, V. et al. Plasma asymmetric dimethylarginine and retinal vessel diameters in middle-aged men. *Metabol. Clin. Exp.* **56**(10), 1305-10 (2007).
15. Sun, C. et al. Quantitative genetic analysis of the retinal vascular caliber: the Australian Twins Eye Study. *Hypertension.* **54**(4), 788-95 (2009).
16. Taarnhøj, N. C. et al. Straight versus tortuous retinal arteries in relation to blood pressure and genetics. *Br. J. Ophthalmol.* **92**(8), 1055-60 (2008).
17. Annunziata, R., Kheirkhah, A., Hamrah, P. & Trucco, E. Scale and curvature invariant ridge detector for tortuous and fragmented structures in *International Conference on Medical Image Computing and Computer-Assisted Intervention*. 588-95 (Springer International Publishing, 2015).
18. Stosic, T. & Stosic, B. D. Multifractal analysis of human retinal vessels. *IEEE Trans. Med. Imaging.* **25**(8), 1101-07 (2006).
19. Wagener, H. P., Clay, G. E. & Gipner, J. F. Classification of retinal lesions in the presence of vascular hypertension. *Trans. Am. Ophthalmol. Soc.* **45,** 57–73 (1947).
20. Patton, N., Aslam, T., MacGillivray, T., Dhillon. B. & Constable I. Asymmetry of retinal arteriolar branch widths at junctions affects ability of formulae to predict trunk arteriolar widths. *Invest. Ophthalmol. Vis. Sci.* **47**(4), 1329-33 (2006).
21. Liew, G. et al. Measurement of Retinal Vascular Caliber: Issues and Alternatives to Using the Arteriole to Venule Ratio. *Invest. Ophthalmol. Vis. Sci*. **48**(1), 52-57 (2007).
22. Lupaşcu, C. A., Tegolo, D. & Trucco, E. Accurate estimation of retinal vessel width using bagged decision trees and an extended multiresolution Hermite model. *Med. Image. Anal.* **17**(8), 1164-8 (2013).
23. Cavinato, A., Ballerini, L., Trucco, E. & Grisan, E. Spline-based refinement of vessel contours in fundus retinal images for width estimation in *Biomedical Imaging (ISBI),* 872-75 (IEEE 10th International Symposium, 2013)
